# Strategies for therapeutic optimization and proactive care in chronic obstructive pulmonary disease (COPD): an Italian expert opinion

**DOI:** 10.1007/s11739-026-04479-8

**Published:** 2026-08-21

**Authors:** Paola Rogliani, Massimo Andreoni, Francesco Dentali, Claudio Micheletto, Andrea Montagnani, Nicola Montano, Alberto Papi, Alessandro Rossi, Pierachille Santus

**Affiliations:** 1 Unit of Respiratory Medicine, Department of Experimental Medicine, University of Rome Tor Vergata, Rome, Italy; 2 Infectious Disease Clinic, Department of Systems Medicine, University of Rome Tor Vergata, Rome, Italy; 3 Department of Medicine and Surgery, Insubria University, Via Ravasi, 2, 21100 Varese, Italy; 4 Respiratory Unit, Integrated University Hospital of Verona, Verona, Italy; 5 Department of Internal Medicine, Hospital Misericordia, 58100 Grosseto, Italy; 6 Department of Clinical Community Sciences, University of Milan, 20122 Milan, Italy; 7 IRCCS Ca’ Granda Foundation, Ospedale Maggiore Policlinico, Milan, Italy; 8 Department of Translational Medicine, Respiratory Medicine Section, University of Ferrara, Ferrara, Italy; 9 Italian College of General Practitioners and Primary Care, Florence, Italy; 10 Division of Respiratory Diseases, “L. Sacco” University Hospital, Università degli Studi di Milano, Milan, Italy

**Keywords:** Chronic obstructive pulmonary disease, COPD, COPD management, Cardiopulmonary risk, Single-inhaler triple therapy, SITT, Exacerbation, Antimicrobial stewardship

## Abstract

Chronic obstructive pulmonary disease (COPD) remains a major public health challenge, characterized by substantial underdiagnosis, high morbidity and mortality, and significant healthcare burden. In Italy, the gap between estimated and diagnosed prevalence highlights the need for improved early detection and proactive management. This expert opinion, developed by a multidisciplinary panel, translates current evidence into practical recommendations tailored to the Italian healthcare context. Patient characterization has moved from a spirometry-centered approach to a proactive, multidimensional model integrating symptom burden, exacerbation history, and cardiopulmonary risk. Early diagnosis should be driven by structured case-finding in primary care and internal medicine, using spirometry for confirmation. Early identification of uncontrolled patients relies on standardized tools, including symptom quantification (CAAT score) and indirect markers like recurrent antibiotic or oral corticosteroid use. Exacerbations are critical events that accelerate both pulmonary and cardiovascular deterioration, identifying a high-risk phase requiring prompt reassessment and optimization. In this context, single-inhaler triple therapy (SITT) is the only pharmacological strategy consistently associated with reduced exacerbations and all-cause mortality in high-risk populations. Timely SITT use may also reduce cardiopulmonary risk. Furthermore, preventing exacerbations limits antibiotic and systemic corticosteroid use, promoting antimicrobial stewardship and mitigating resistance. This expert opinion emphasizes the need for integrated care pathways, standardized clinical tools, and multidisciplinary coordination to overcome therapeutic inertia. A shift toward early identification, timely intervention, and prevention-oriented strategies is essential to improve outcomes and reduce the systemic burden of COPD.

## Introduction: epidemiological landscape and unmet needs

### Disease burden in Italy

Chronic Obstructive Pulmonary Disease (COPD) represents a major public health challenge worldwide. In Italy, prevalence is estimated at 5.6% of the adult population, affecting over 3.5 million individuals, and accounts for more than 55% of deaths due to respiratory conditions [1]. However, administrative data reports a diagnosed prevalence of 2.88%, revealing a substantial underdiagnosis and suggesting that many patients are missing the window for early intervention.

National outcome analyses highlight marked regional heterogeneity in hospitalization rates, indicating disparities in diagnostic pathways and care organization [1, 2]. Moreover, fragmented data, incomplete exacerbation capture, and the lack of national registries likely underestimate healthcare utilization [3]. Most deaths (79.3%) occur outside hospital settings, underscoring a substantial hidden community burden.4 Among in-hospital deaths, cardiovascular causes (40.7%) exceed respiratory causes (31.4%), reflecting the systemic nature of COPD and importance of concomitant chronic conditions.4 Mortality risk peaks immediately after exacerbation: hazard ratios for all-cause death reach 15.13 within 1–7 days and remain elevated within 15–30 days [4]. Thirty-day posthospitalization mortality exceeds 13% in Italy [1], and excess risk persists up to one year, with nearly 18% mortality within 12 months after discharge [4, 5]. Hospitalization therefore represents a prognostic inflection point marking sustained cardiopulmonary vulnerability.

### Evolution of the clinical paradigm

The Global Initiative for Chronic Obstructive Lung Disease (GOLD) framework has moved beyond a purely spirometric classification toward a multidimensional model integrating symptoms, quality of life (QoL) and exacerbation risk [6]. COPD is increasingly recognized as a chronic condition closely linked to multimorbidity, particularly cardiovascular [6].

In light of persistent gaps in diagnosis and treatment optimization, this expert opinion, developed by a multidisciplinary panel of pulmonologists, internists, general practitioners (GPs) and infectious disease specialists, aims to adapt international evidence to the Italian context, promoting earlier identification, timely initiation of triple therapy, and a structured approach to cardiopulmonary risk management.

## Early diagnosis of COPD: case-finding and diagnostic strategies

### Frontline identification in primary care and internal medicine

Early diagnosis of COPD often begins outside specialist respiratory settings. GPs and internal medicine physicians play a central role in the early identification of individuals with suspected COPD. Clinical suspicion should extend beyond current smokers to include former smokers, individuals with occupational exposures, and residents in high-pollution areas [1, 6]. Through structured symptom assessment, review of exacerbation proxies, and timely referral for spirometry, primary care can intercept the large “submerged” population of undiagnosed patients.

This proactive case-finding approach is equally relevant in hospital settings. Expanding the knowledge of the importance of a proper diagnosis to Internists and establishing specific referral pathways may facilitate the identification of previously unrecognized COPD among patients hospitalized for cardiovascular or other comorbid conditions [7].

### Diagnostic confirmation and evolving diagnostic criteria

The formal diagnosis of COPD has traditionally relied on demonstrating persistent airflow obstruction at spirometry (postbronchodilator FEV₁/FVC < 0.70) in symptomatic subjects exposed to risk factors. However, spirometry should be considered primarily a tool for diagnostic confirmation rather than for guiding therapeutic decisions, which are instead driven by symptom burden and exacerbation history [6]. Patients with respiratory symptoms or structural abnormalities on imaging may experience outcomes comparable to those with established airflow obstruction [8, 9]. Chest computed tomography (CT) can reveal emphysema, airway wall thickening, or air trapping even in individuals with preserved FEV₁, supporting a broader integration of symptoms, imaging, and functional assessment [6]. However, current evidence supporting pharmacological treatment is primarily derived from populations with spirometrically confirmed COPD.

Pre-bronchodilator simplified spirometry has shown high diagnostic accuracy while reducing examination time, supporting a pragmatic strategy for earlier identification of high-risk individuals.

## Early identification of the uncontrolled COPD patient

### Clinical assessment tools and symptom burden

GOLD 2026 recommends validated tools such as the Chronic Airways Assessment Test (CAAT) for grading symptom burden and guiding clinical evaluation [6]. A CAAT score ≥ 10 identifies at least moderate symptom burden within the GOLD-ABE initial treatment framework [6], warranting therapeutic reassessment [10]. In addition, routine audits of electronic medical records (EMR) to identify patients lacking a recent CAAT evaluation (e.g., within the last 6 months) or presenting with persistently elevated CAAT scores can prompt early follow-up visits and proactive treatment optimization.

While not diagnostic, these measures offer accessible indicators of clinically relevant impairment. Systematic integration of CAAT scoring and basic functional assessment transforms subjective symptom reporting into measurable parameters, facilitating earlier recognition of instability.

### Indirect identification and exacerbation proxies

A substantial proportion of exacerbations remain unreported or are coded under nonspecific diagnoses, contributing to the “clinical submerged” burden of the disease. Indirect identification strategies based on prescription data can help uncover hidden disease activity.

Prescriptions of antibiotics and/or oral corticosteroids (OCS) are conventionally used to define moderate exacerbations [6, 11]. In a large population-based cohort, increasing exacerbation frequency was associated with a higher risk of subsequent exacerbations and mortality, including cardiovascular mortality, even after a single moderate event [11].

Structured “active case finding” through EMR review, identifying patients with repeated antibiotic or OCS prescriptions in the previous 12 months, represents a scalable approach to detect uncontrolled or undiagnosed COPD [10]. In smokers or former smokers, recurrent antibiotic-treated “bronchitis” should systematically prompt spirometric evaluation [6]. Prescription history review thus becomes a pragmatic tool to intercept high-risk patients in routine practice.

### Hospitalization as a diagnostic opportunity

Given the high postdischarge vulnerability, with 34.6% of patients experiencing a severe cardiopulmonary event and 18.2% dying within 1 year [4], standardized discharge checklists should be implemented as a quality priority. These clinical tools ensure systematic diagnostic confirmation (including spirometry at follow-up, if missing), symptom assessment, risk stratification, inhaler technique verification and appropriate referral for follow-up [6, 10]. Unfortunately, the opportunity for therapeutic revision and step-up, after these sentinel events, is frequently missed.

## Therapeutic optimization: timing and prescribing appropriateness

### The rationale for triple therapy

Recent evidence reinforces the concept that exacerbation prevention must be a primary therapeutic objective. GOLD 2026 emphasizes that even a single moderate exacerbation significantly increases future risk and mortality [6, 11, 12], supporting the concept of “Zero Exacerbations” as a clinically meaningful target.

Triple therapy is the only inhaled strategy supported by randomized trials demonstrating mortality reduction versus dual bronchodilation in high-risk COPD populations. In ETHOS, budesonide/glycopyrrolate/formoterol reduced all-cause mortality compared with long-acting muscarinic antagonist/long-acting β₂-agonist (LAMA/LABA) [13, 14]. In IMPACT, fluticasone furoate/umeclidinium/vilanterol showed similar mortality benefit, consistent in Western European populations [15, 16]. Triple therapy also consistently reduces moderate-to-severe exacerbations versus dual regimens across ETHOS, IMPACT, TRILOGY, TRIBUTE and TRINITY [14, 15, 17–19].

Despite the robust evidence supporting single-inhaler triple therapy (SITT) in reducing exacerbations and mortality [20–24], SITT adoption remains limited. The PROMETHEUS Italy model estimates that optimized SITT use could prevent approximately 40,000 deaths and 109,000 severe exacerbations over 10 years [25].

Timing is critical; real-world data show that SITT initiation within 30 days after an exacerbation significantly reduces subsequent exacerbations and cardiopulmonary events compared with delayed escalation [22]. These data support moving beyond a strictly stepwise model in high-risk patients.

### Barriers to the adoption of triple therapy

Despite the strong clinical evidence and guideline endorsement supporting triple therapy, its adoption in routine practice remains suboptimal, particularly outside specialist respiratory settings. Multiple barriers continue to limit timely therapeutic escalation, reflecting educational, cognitive, organizational, and regulatory challenges within healthcare systems. These barriers contribute to delayed treatment optimization and persistent use of suboptimal therapeutic regimens; the main factors contributing to therapeutic inertia are summarized in Table 1.

**Table 1:** Multilevel barriers to the adoption of inhaled triple therapy in COPD

Domain	Key barrier	Clinical consequence
Educational/cultural	Limited awareness of updated GOLD recommendations outside specialist settings	Delayed escalation; persistent use of ICS/LABA (24–27% in GP) despite guideline shift [6]
	COPD is perceived as less urgent than cardiovascular comorbidities	Therapeutic inertia and under-optimization
Clinical perception	Exacerbations interpreted as isolated infectious events	Failure to escalate therapy after moderate events
	Insufficient recognition of exacerbation–mortality link	Underestimation of future cardiopulmonary risk [11, 25]
Organizational	Fragmented hospital–community pathway	Lack of structured postdischarge optimization [5]
	Discharge without maintenance or escalation to triple therapy	Increased risk of readmission and mortality
	Limited availability of spirometry in Internal Medicine wards	Missed or delayed diagnosis during acute events [1]
Regulatory/structural	Historical prescribing restrictions	Cultural hesitation despite the removal of barriers
	Implementation of Nota 99 with bureaucratic bottlenecks	Temporary contraction of maintenance prescriptions

COPD, chronic obstructive pulmonary disease; GOLD, Global Initiative for Chronic Obstructive Lung Disease; ICS, inhaled corticosteroid; LABA, long-acting β₂-agonist

### Clinical management by care setting

The key principles for proactive patient identification and disease optimization, differentiated by community and hospital care settings, are summarized in Table 2.

**Table 2:** COPD clinical management and therapeutic escalation by care setting

Care Setting	primary goal	Target population/risk profile	Clinical implications
Community	Identifying the symptomatic patient and preventing the first severe event	Patients exhibiting a high symptom burden (CAAT ≥ 10) or relying on recurrent antibiotic/OCS use	These indicators signal increased disease burden and exacerbation risk. Structured clinical reassessment and treatment review should be considered in accordance with current recommendations [6, 10, 20]
Hospital	Mandatory clinical review at discharge	Patients hospitalized for respiratory events or COPD exacerbations; patients discharged after acute respiratory deterioration [5]	Hospitalization identifies patients at particularly high short- and long-term risk. Discharge represents a critical opportunity to review treatment, ensure appropriate follow-up and continuity of care

CAAT, Chronic Airways Assessment Test; COPD, chronic obstructive pulmonary disease; OCS, oral corticosteroids

## Systemic implications and cardiopulmonary protection

### Pathophysiology of exacerbation: the “systemic trigger”

COPD exacerbations should be interpreted not as isolated respiratory deteriorations but as systemic biological “stress tests” capable of triggering distal organ injury. Acute events are characterized by marked systemic inflammation (elevated interleukin-6, C-reactive protein, and fibrinogen) together with sympathetic activation, endothelial dysfunction, and a prothrombotic state [7, 10, 26]. This systemic condition destabilizes atherosclerotic plaques, promoting acute atherothrombosis and ischemic events [22, 26]. Simultaneously, hypoxemia, tachycardia, and dynamic hyperinflation increase myocardial oxygen demand while impairing ventricular filling, predisposing to ischemia, arrhythmias, and heart failure decompensation [7, 26]. These mechanisms define cardiopulmonary risk—the integrated risk of myocardial infarction, stroke, heart failure, severe exacerbations, and death [7, 26]. In this framework, exacerbations function as accelerators of both pulmonary and cardiovascular deterioration.

### Cardiovascular risk: the “vulnerability window”

Real-world data identify a critical vulnerability window immediately following exacerbation. In the Italian EXACOS-CV cohort (> 200,000 patients), risk of severe cardiovascular events peaked within 7 days, particularly for heart failure, and remained elevated for up to 1 year [4]. Similar temporal patterns were observed internationally [27, 28]. In the GULP study, 13% of patients died over 3 years; mortality was strongly associated with heart failure and ischemic heart disease, and severe exacerbations were more frequent among deceased patients [12]. Postdischarge cohorts confirm high event rates (34.6%) and 1-year mortality of 18.2% [5, 29]. The immediate postexacerbation phase, especially after hospitalization, therefore represents a high-risk period requiring intensified follow-up and therapeutic optimization [4, 5, 27].

### Active pharmacological protection: from symptom control to risk reduction

Randomized trials have demonstrated that inhaled triple therapy reduces exacerbations [14, 15, 17–19] and all-cause mortality in high-risk COPD populations [13–15]. Post-hoc analyses from major trials indicate reductions in composite cardiopulmonary outcomes, suggesting that attenuation of exacerbation frequency may translate into indirect cardiovascular protection [13].

Real-world evidence reinforces this suggestion. In SKOPOS-MAZI, SITT was associated with lower mortality and fewer severe cardiopulmonary events compared with multiple-inhaler therapy [23]. Early postexacerbation initiation further reduced subsequent events [5–22]. At a population level, PROMETHEUS Italy projected substantial mortality reductions with optimized SITT adoption, underscoring the potential systemic impact of guideline-concordant prescribing [25].

Beyond inhaled therapy, cardiovascular disease must be treated as a modifiable trait. Systematic risk assessment (Systematic COronary Risk Evaluation 2-SCORE2, QRISK4) and guideline-directed management of heart failure, ischemic heart disease, hypertension, dyslipidemia, and atrial fibrillation are essential [7, 10]. Exacerbation prevention and cardiovascular optimization act synergistically in modifying long-term risk [7, 26].

Optimal management of the cardiopulmonary patient requires overcoming outdated concerns regarding drug interactions. Cardioselective beta-blockers are safe and should not be withheld when indicated [6, 7]. Sodium–glucose cotransporter-2 (SGLT2) inhibitors, central to heart failure therapy, are being explored for potential anti-inflammatory and metabolic effects within the cardiopulmonary continuum [7, 10].

## Antimicrobial resistance (AMR) stewardship and steroid-sparing strategies

### Optimization of inhaled therapy and prophylaxis: the primary stewardship

In patients with COPD, frequent exacerbations represent a major driver of antibiotic and systemic corticosteroid use. Consequently, strategies aimed at preventing exacerbations play a central role in antimicrobial stewardship. In this perspective, exacerbation prevention becomes a key upstream intervention to reduce antibiotic exposure and limit the development of antimicrobial resistance (AMR). The panel, therefore, emphasizes that optimal long-term disease management should include AMR containment strategies.

#### High antibiotic consumption and resistance burden

Patients with COPD receive antibiotics approximately three times more frequently than individuals without COPD [30]. Italian real-world data confirm this disproportionate exposure, with 59% of COPD patients receiving antibiotics annually compared with 41% of the general population [31]. Up to 70% of exacerbations are infection-related, and about half involve bacterial pathogens [31]. Repeated antibiotic exposure generates selective pressure favoring resistant strains, while the COPD airway environment, characterized by chronic inflammation, impaired mucociliary clearance, and biofilm formation, facilitates persistent colonization [30, 32]. Common pathogens include *Haemophilus influenzae, Streptococcus pneumoniae, Pseudomonas aeruginosa, Staphylococcus aureus* (including Methicillin-Resistant *Staphylococcus aureus*), and *Acinetobacter baumannii*, many exhibiting clinically relevant resistance patterns, particularly in hospitalized patients [30, 32].

#### Preventive stewardship: optimization of maintenance therapy

Preventing exacerbations represents the most effective antimicrobial stewardship strategy in COPD. Each avoided exacerbation translates into fewer antibiotic prescriptions and reduced systemic corticosteroid exposure. Timely optimization of maintenance therapy, particularly with SITT, plays a critical role in this context. Randomized trials consistently showed that inhaled triple therapy significantly reduces moderate and severe exacerbations compared with dual therapy [14, 15]. By decreasing exacerbation frequency, SITT indirectly reduces the need for antibiotic and OCS treatments, thereby lowering antimicrobial pressure.

From a public health perspective, optimal COPD control therefore contributes not only to improved respiratory outcomes but also to the containment of AMR.

#### Vaccination strategy as a core component of stewardship

Comprehensive vaccination coverage is a cornerstone of primary prevention. GOLD 2026 recommends routine immunization to reduce infection-triggered exacerbations [6]. Current consensus supports influenza, pneumococcal (PCV20/PCV21), SARS-CoV-2, pertussis, herpes zoster, and respiratory syncytial virus (RSV) vaccination in older adults [6, 32]. By preventing viral and bacterial respiratory infections, vaccination reduces exacerbations and consequently antibiotic and steroid use, functioning as an integral component of antimicrobial stewardship [32].

### Evolved number needed to treat (NNT): incorporating “avoided antibiotic/steroid cycles”

To capture the full public health value of COPD therapy, experts propose an “evolved” interpretation of the *Number Needed to Treat* (NNT) that includes avoided antibiotic and systemic corticosteroid cycles. Each prevented exacerbation represents not only clinical stabilization but also reduced antimicrobial exposure and lower systemic steroid burden—important given the metabolic, cardiovascular, and infectious risks associated with repeated steroid courses. Moreover, antimicrobial resistance can be viewed as a downstream consequence of poorly controlled COPD.

By preventing exacerbations, optimized inhaled therapy reduces colonization with multidrug-resistant organisms, limits resistant infection-related hospitalizations, and decreases transmission within healthcare settings [32]. Integrating avoided antibiotic/steroid cycles into efficacy evaluation shifts the perspective from individual exacerbation prevention to population-level AMR containment, positioning optimal COPD management as a form of primary antimicrobial stewardship.

## Conclusions: consensus statements and health policy perspectives

### Synthesis of clinical recommendations

Overcoming therapeutic inertia requires a shift from reactive to proactive COPD management. The panel identified three priority cognitive automatisms for daily practice.

First, clinical suspicion must be standardized. The association “smoker (or former smoker) + chronic dyspnea = suspected COPD” should systematically trigger spirometry to uncover the large “submerged” population of undiagnosed or misclassified patients. Suspicion should extend to former smokers and individuals with environmental or occupational exposure.

Second, “exacerbation equals risk” must become embedded in clinical reasoning. Exacerbations are not isolated infectious episodes but markers of increased risk of future exacerbations, along with mortality and cardiopulmonary events [7, 12]. Even a single moderate exacerbation warrants therapeutic reassessment and possible escalation according to GOLD recommendations [6].

Third, structured assessment should be routine. Systematic CAAT evaluation and review of prescription history, particularly recurrent antibiotic and oral corticosteroid use, must be integrated into every visit [10]. These simple tools allow early identification and anticipation/intensification of treatment and preventive measures (e.g., vaccination).

### Operational tools for implementation

Translating consensus into practice requires structured and reproducible organizational tools. Hospitalization for COPD exacerbation should be considered a clinical red flag and a mandatory optimization point. Structured discharge checklists or bundles can ensure confirmed diagnosis, therapeutic reassessment, including consideration of SITT in eligible patients, and scheduled follow-up [10].

In primary care, proactive case-finding should be supported by systematic medical record audits. The implementation of electronic alerts to flag patients with CAAT > 10, missing recent assessments, or repeated antibiotic/OCS prescriptions may support proactive intervention and reduce preventable exacerbations.

To assess real-world impact, healthcare systems should monitor *key performance indicators*, including annual exacerbation rate per patient; number of avoidable antibiotic and OCS courses as a proxy for disease control; hospitalization and emergency department visit rates for respiratory causes; and the proportion of eligible uncontrolled patients appropriately initiated or escalated to SITT [25]. Monitoring these indicators may support clinical governance and align local practice with evidence-based standards.

### Institutional call to action

Addressing the COPD burden requires coordinated engagement beyond the clinical setting. COPD should be fully integrated into national prevention and chronic disease plans to ensure equitable access to spirometry for diagnostic confirmation, structured pathways, and optimized pharmacological care across regions.

Comprehensive vaccination strategies, including influenza, pneumococcal, RSV, SARS-CoV-2, pertussis, and herpes zoster vaccines, must be embedded within COPD care pathways, consistent with GOLD recommendations [6,  32]. By preventing infection-driven exacerbations, vaccination supports both individual protection and antimicrobial stewardship. A broader cultural shift is needed to reposition COPD as a severe yet treatable systemic disease, comparable in urgency to cardiovascular conditions [7].

Finally, optimized maintenance therapy and timely SITT initiation in eligible patients should be recognized as strategic tools for AMR mitigation, reducing unnecessary antibiotic exposure and contributing to broader public health goals.

### Closing remarks

**Fig. 1:**
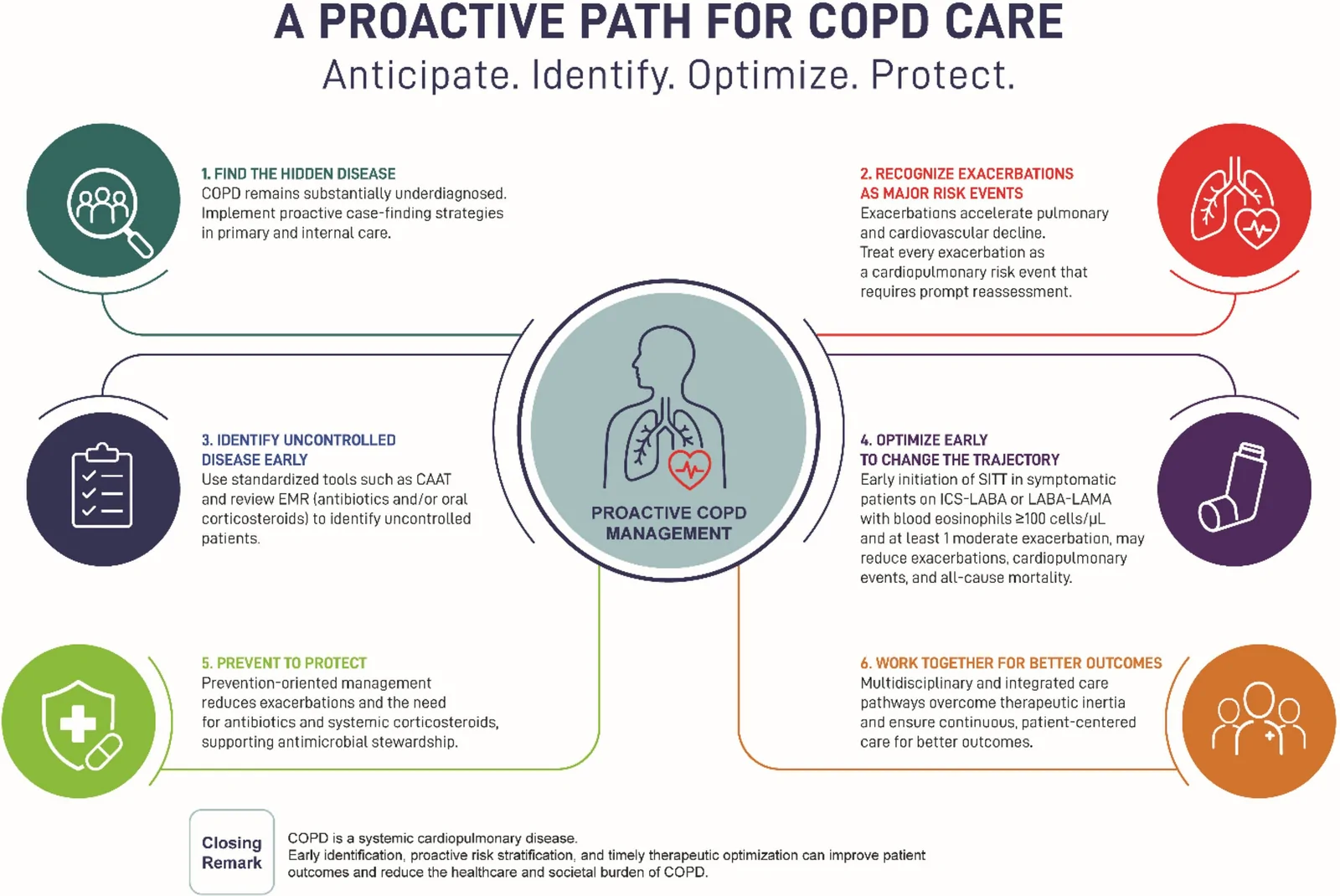
Proactive COPD management model: from early identification to prevention-oriented care. CAAT, Chronic Airways Assessment Test; COPD, chronic obstructive pulmonary disease; EMR, electronic medical record; ICS, inhaled corticosteroid; LABA, long-acting β₂-agonist; LAMA, long-acting muscarinic antagonist; OCS, oral corticosteroids; SITT, single-inhaler triple therapy

COPD should no longer be viewed as an exclusively respiratory disease, but rather as a systemic condition affecting different organs and systems (e.g. the cardiovascular system), particularly during and after acute exacerbation episodes, requiring early identification, proactive risk stratification, and timely therapeutic optimization. Moving from a reactive to a prevention-oriented model of care may improve clinical outcomes by reducing exacerbations, cardiopulmonary complications, and mortality, while also limiting avoidable antibiotic and systemic corticosteroid exposure and supporting antimicrobial stewardship. The implementation of standardized assessment tools, integrated care pathways, and timely initiation of evidence-based therapies represents a key opportunity to reduce therapeutic inertia and close the gap between current evidence and real-world practice. Ultimately, a proactive and multidisciplinary approach is essential to reduce the individual, societal, and healthcare burden of COPD. The key principles of the proposed proactive COPD management model are summarized in Fig. 1.

## Data availability statement

NA.
